# Cancer prevention as biomodulation: targeting the initiating stimulus and secondary adaptations

**DOI:** 10.1111/j.1749-6632.2012.06736.x

**Published:** 2012-10-10

**Authors:** Priscilla A Furth

**Affiliations:** Departments of Oncology and Medicine, Lombardi Comprehensive Cancer Center, Georgetown UniversityWashington, DC and Department of Nanobiomedical Science and WCU Research Center of Nanobiomedical Science, Dankook UniversityChungnam, Korea

**Keywords:** cancer prevention, biomodulation, hit and run oncogenesis, viral oncogenesis, hormonal oncogenesis

## Abstract

In a medical sense, biomodulation could be considered a biochemical or cellular response to a disease or therapeutic stimulus. In cancer pathophysiology, the initial oncogenic stimulus leads to cellular and biochemical changes that allow cells, tissue, and organism to accommodate and accept the oncogenic insult. In epithelial cell cancer development, the process of carcinogenesis is frequently characterized by sequential cellular and biochemical adaptations as cells transition through hyperplasia, dysplasia, atypical dysplasia, carcinoma *in situ*, and invasive cancer. In some cases, the adaptations may persist after the initial oncogenic stimulus is gone in a type of “hit-and-run” oncogenesis. These pathophysiological changes may interfere with cancer prevention therapies targeted solely to the initial oncogenic insult, perhaps contributing to resistance development. Characterization of these accommodating adaptations could provide insight for the development of cancer preventive regimens that might more effectively biomodulate preneoplastic cells toward a more normal state.

## Introduction

To some extent, all therapeutic interventions could be considered biomodulation, as they are all directed toward a change in the individual's pathophysiology at both cellular and systemic levels. For the purposes of discussion here, *biomodulation* is defined in medical terms as a change in cells or tissue in response to a pathologic or therapeutic stimulus. For example, it has been applied to specific therapies for kidney disease, that is, the impact of freeze-dried *Lactobacillus acidophilus* on the bacterial overgrowth syndrome occurring in end-stage kidney disease.^1^ In oncology, the broad concept of biomodulation is incorporated into cancer evolution and therapy and many of the approaches and techniques developed for systems biology are used within the context of oncological investigations to characterize specific “biomodulating” events at different stages of cancer evolution or therapy. In cancer therapy, this type of approach has been validated. For example, the addition of agents to previously defined standard chemotherapeutic regimens is described as biomodulating the therapeutic response.^2^–^9^ Investigations have validated the same type of approach for improving response to radiotherapy,^10^,^11^ laser irradiation,^12^–^16^ and application of Chinese medicine to cancer care.^17^

**Figure 1:**
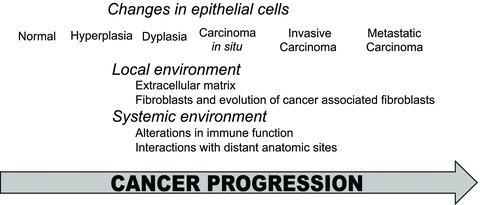
Biomodulation of cancer tissue occurs in both epithelial and stromal compartments throughout cancer evolution. Changes in the epithelial and stromal compartments occur as epithelial cells transition from normal through hyperplasia, dysplasia, carcinoma *in situ*, invasive cancer, and metastasis. The engagement of epithelial cells in crosstalk with the local and systemic environments during cancer progression results in their biomodulation. Different biomodulatory events can occur at different stages of cancer progression (reviewed in Refs. 18–20).

**Figure 2:**
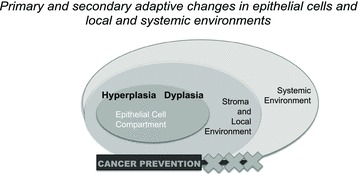
Targets for biomodulation in cancer prevention. Targets for biomodulation in epithelial cancer prevention can include the epithelial cells themselves as well as factors in the local stromal and systemic environments (reviewed in Refs. 21 and 22).

Evolution of cancer in epithelial tissues frequently develops as a sequence of coordinated changes in both the epithelial and stromal compartments (Fig. 1). Biomodulatory changes occur throughout the process and can be both similar and different in preneoplastic, hyperplastic, and dysplastic tissue, invasive cancer, and metastases. The field of cancer prevention seeks to interrupt cancer development before it reaches either the carcinoma *in situ* or invasive stages. The value of considering cancer prevention in the context of biomodulation would be to conceptually encompass the therapeutic approach as both an assault on the initiating event as targeted therapy, as well as an attack on the secondary adaptations that a preneoplastic cell, and surrounding local stromal and systemic environments, may have made to survive or adapt to that initiating stimulus (Fig. 2). The probability of epithelial cancer development can be altered by germ-line genetic predisposition such as mutations in breast cancer 1, early onset (*BRCA1*), breast cancer 2, early onset (*BRCA2*), phosphatase and tensin homolog (*PTEN*), and tumor protein p53 (*TP53*). It is possible that specific biomodulatory events accompany epithelial cancer progression in these cases. Identification of these events may lead to more targeted chemopreventive approaches for individuals carrying these mutations. Some types of sporadic cancer development are also characterized by genetic changes, sometimes in the same genes found with familial germ-line predisposition. It will be important to determine whether the types of biomodulatory changes that occur when cancer develops secondary to a germ-line risk factor are the same as those that may occur when the mutation occurs in somatic cells as part of sporadic cancer development. It is quite possible that genetic background, both germ-line and somatic, will influence the response to specific biomodulatory approaches.

This short review concentrates on what type of molecular changes might occur in an epithelial cell as secondary cellular adaptations that occur over time following a carcinogenic stimulus, to determine if they are disease drivers, and to test if they can be recognized before therapy targeted to the initial stimulus or whether they only become apparent after the initiating stimulus has been removed. It is possible that cancer prevention approaches that target both disease-initiating stimuli and secondary adaptations would more efficiently effect disease reversal.

## Identifying cellular adaptations that occur over time in cells exposed to a carcinogenic stimulus

Cancer development is not a unitary process but instead is executed by different processes. A relatively direct mechanism of cancer development and maintenance is “oncogene addiction.” In this situation the growth and survival of the cancer cell is dependent upon maintenance of a cancer causing stimulus, such as a defined oncogene,^23^–^25^ miRNA,^26^ or immune-mediated mechanism.^27^ Less direct is the multistep mechanism termed “hit-and-run” oncogenesis, where the initiating stimulus is gone but the tissue continues to progress toward, or is maintained as, cancer. Hit-and-run oncogenesis is most frequently considered in the context of virally induced cancers.^28^ The idea that cancer progression can persist in the absence of initiating viral oncoprotein expression is supported by laboratory models in which a transformed cellular phenotype persists despite loss of viral oncoprotein expression for adenoviruses,^29^,^30^ herpesviruses,^31^,^32^ and the polyomavirus Simian virus 40 (SV40) large T antigen (TAg).^33^–^37^ Hit-and-run–mediated pathophysiology has been suggested as a pathway for polyomavirus in human brain tumors and mesotheliomas,^38^–^40^ JC virus in colorectal cancer,^41^ papillomaviruses in Schneiderian inverted papillomas,^42^ and Hepatitis B in hepatomas.^43^ The challenge in accepting a hit-and-run mechanism is that, by definition, the inciting event is lost in the cancer that develops, essentially precluding fulfillment of the fourth of Koch's postulates.^44^ Induction of chromosomal instability^45^ and epigenetic reprogramming^46^ have been suggested as mechanisms that maintain transformed cell growth in the absence of the initiating virus. Other groups have suggested that in fact a small proportion of virally infected cells persist and secrete paracrine growth factors that maintain abnormal growth of a larger majority of uninfected cancer cells.^47^ Duration of exposure to an oncogene may play a role in the development of secondary oncogenic adaptations. For example, longer durations of infection are associated with a higher risk of neoplastic transformation in papillomavirus-induced cervical cancer;^48^ liver cancer incidence increases as the duration of Hepatitis C infection extends past 25 years;^49^ and in a mouse model of TAg-induced salivary epithelial cell dysplasia, abnormal activation of the cyclin-Cdk-Rb pathway by prolonged exposure to the viral oncoprotein maintains the dysplastic phenotype when TAg is downregulated.^37^ These *in vivo* phenomenon can be modeled in some tissue culture cells *in vitro*. For example, long-term exposure to estrogen can induce persistent changes in gene expression in the estrogen receptor alpha (ERα)-positive MCF7 cell line.^50^

## Determine if adaptive change predicts response to therapy targeted to primary oncogenic stimulus

Experimentally, a conditional system, such as the tetracycline responsive gene expression system^51^ in which oncogene expression can be directly turned on and off in experimental animals, is helpful in establishing experiments that can test whether or not an oncogene induces oncogene addiction^52^–^58^ or a hit-and-run type of oncogenesis that leaves mutations or other genetic changes behind that then maintain carcinogenesis.^33^,^36^,^37^,^54^,^55^,^59^,^60^ In Myc-induced oncogenesis, investigations have found that p53 or thrombspondin-1^60^ and CD4^+^ T cells^61^ can be required for regression following loss of Myc, with CD4^+^ T cells playing a role in regression following loss of Breakpoint cluster region-Abelson (BCR-ABL) as well.^61^ Activating mutations in K-ras2 can contribute to maintenance of Myc-induced carcinogenesis independent from expression of the *myc* oncogene.^54^,^55^,^59^ In lung tumors and lymphomas induced by K-ras(G12D) or Myc, levels of phosphorylated extracellular signal-regulated kinase (Erk) 1 and 2, Akt1, signal transducer and activator of transcription (STAT) 3/5, and p38 may predict whether or not a cancer will regress when the initiating oncogene is downregulated.^56^ In TAg-induced salivary dysplasia, upregulated expression of phosphorylated retinoblastoma (pRb) and transcription factor Dp-1 predict that dysplasia will reverse when TAg is downregulated, whereas low expression levels portend nonreversal.^37^

## Removal of an oncogenic stimulus can result in the appearance of previously unnoticed pathophysiology

Conditional experimental systems also enable examination of cellular and biochemical changes that occur after the initiating oncogenic stimulus has been downregulated. In the case of TAg expression in striated duct cells in the submandibular salivary gland, dysplasia reverses when TAg is downregulated at four months of age but persists when TAg is downregulated at seven months of age.^33^,^36^,^37^ Before TAg downregulation at the two different ages, there are relatively few differences in gene expression as detected by cDNA array analysis,^36^ while elevated levels of pRB and Dp-1 in four-month-old mice have been the only differences on the protein level identified to date. In contrast, within days of TAg downregulation multiple differences in protein expression levels and extent of phosphorylation appear.^37^ Phosphorylated Rb levels increase in the seven-month-old mice but decrease in the four-month-old mice. p21 and p27 rise in four-month-old mice, paralleling the cell cycle arrest observed at that age when TAg is downregulated, but remain low in the seven-month-old mice that exhibit persistent cell cycle activation. Cdk4, Cdk6, and cyclin D1 levels rise initially at both ages after TAg downregulation, but this rise is reversed in the four-month-old mice, whereas it persists in the seven-month-old mice. Dp-1 levels fall in the four-month-old mice but increase in the seven-month-old mice. These experiments illustrate that underlying cellular and biochemical pathophysiology may only be revealed when an initiating oncogenic stimulus is removed. There are analogies in the response of breast cancer cells to tamoxifen, a mixed ERα antagonist/agonist used for primary and secondary prevention of ERα-positive breast cancer.^62^ Mucin4, which can reactivate the HER2 pathway, is upregulated in breast cancer cells following combined treatment with tamoxifen and targeted HER2 therapy.^63^ Moreover, tamoxifen-resistant cells are reported to demonstrate upregulation of a variety of proteins including methylated-DNA-protein-cysteine *S*-methyltransferase (MGMT),^64^ increased expression or phosphorylation of NF-κB pathway proteins p50, RelB, and p65,^65^ and downregulation of miRNA (miR)-375.^66^ It is possible that changes like these found in breast cancer cells can also occur in preneoplastic cells, compromising the impact of tamoxifen as a preventive. Measuring the cellular and biochemical changes that occur after targeting an oncogenic stimulus can provide a fuller understanding of the underlying pathophysiology in preneoplasia, as well as cancer, and may be useful in predicting which resistance pathways will develop.

## Elucidating the pathological changes that are disease-driving

In the case of TAg-induced age-dependent reversible and irreversible hyperplasia and dysplasia, there are multiple molecular dissimilarities.^37^ Identification of the disease-driving molecular aberrations requires developing experimental approaches that can isolate and identify the key driver. In this case, abnormal activation of the Cdk4/6 pathway, and Cdk4 in particular, was found to be the culprit, but it was identified only through a series of experiments.^37^ First, related pathway molecules Cdk6 and cyclin D1 were eliminated as essential drivers by themselves because both molecules were significantly downregulated by administration of rexinoid X receptor (RxR) and peroxisome proliferator-activated receptor gamma (PPARγ) agonists, but dysplasia was not reversed. Successful reversal occurred only with the orally available Cdk4/6 inhibitor (PD-0332991), which downregulated Cdk4/Cdk6 and cyclin D1. This illustrated that Cdk4 is an essential driver of the dysplastic phenotype. Other experiments showed that the differences in p21, p27, and phosphatase 2A expression levels were immaterial in dysplasia reversal.^36^,^37^

Similar to the salivary gland experimental system, loss of cell cycle checkpoints also occurs during breast cancer progression. Some endocrine-resistant breast cancers in women are reported to exhibit higher levels of pRb, consistent with loss of cell cycle control.^67^ The same orally available Cdk4/6 inhibitor (PD-0332991), used successfully to reverse previously irreversible salivary dysplasia,^37^ has been suggested as an aide to antihormone therapy for ER-positive breast cancer.^67^–^70^ Activation of Cdk4/6 can occur secondary to estrogen pathway activation. But different primary drivers, even in the same pathway, may stimulate different secondary adaptations. For example, in genetically engineered mouse models, aromatase overexpression, but not ERα overexpression, stimulates increased levels of Cdk2.^71^ In preclinical studies, Cdk1/2 inhibitors have been shown to have activity in anti-estrogen–resistant breast cancer cells.^72^ As discussed earlier, levels of Erk1/2, Akt1, and STAT3/5 are possible predictors of reversal (versus nonreversal) when K-ras (G12D) or Myc are downregulated in lung tumors and lymphomas of experimental mice.^56^ Levels of Erk1/2 and STAT3/5 phosphorylation are significantly increased in mammary epithelial cell preneoplasia triggered by either ERα or aromatase overexpression, whereas phosphorylated Akt is increased in the aromatase over-expressing mice.^71^ Both of these mouse models demonstrate resistance to the ERα downregulator ICI 182, 780 (Faslodex®, AstraZeneca Pharmaceuticals LP, Wilmington, DE)^73^ when it is used as an agent to promote regression of preneoplasia, but the percentage of mice demonstrating resistance is higher in the aromatase overexpressing mice.^71^ It is possible that the higher degree of resistance found in the aromatase overexpressing mice is related to the fact that these mice demonstrate increased expression levels of Cdk2 and phosphorylated Akt, whereas the ERα overexpressing mice do not. Experiments analogous to those that identified Cdk4 as a primary driver in the TAg-induced salivary dysplasia could identify which, if any, of the identified molecular aberrations might be secondary adaptations contributing to antihormone resistance.

## Cancer prevention approaches that target disease-initiating stimuli and secondary adaptations

The message from experimental studies of cancer progression and reversal is that when oncogene addiction is not the case, secondary pathophysiological adaptations induced by oncogene exposure may need to be identified and targeted for successful therapy (Table 1). Studies in salivary dysplasia indicate that these changes can occur not only in cancer cells but in preneoplastic cells as well. In some cases, preventive therapy may be more appropriately configured as “biomodulation” therapy that not only aims to disarm the initial disease-causing stimulus but also targets the secondary adaptations that can occur as a cell and tissue adapt to an abnormal growth stimulus.

**Table 1:** Summary of published examples of secondary adaptations that are associated with disease maintenance following downregulation of an initiating cancer stimulus

After downregulation/inactivation/antagonism of initiating stimulus							
Initiating stimulus	disease maintenance after down-regulation of initiating stimulus	Accompany disease maintenance	Experimentally defined as *disease-sustaining* secondary adaptations	Disease regression factors	Experimental system	Tissue	References
SV40TAg	Low levels of pRb and Dp-1	Increased levels of Dp-1, Cdk4, Cdk6, cyclin D1, and phosphatase 2A; low p21 and p27	Increased levels of Cdk4		Genetically engineered mice	Submandibular salivary gland	37
Myc	Increased Erk1/2, Akt1, Stat3/5, p38 phosphorylation	Kras2 activating mutation	Kras2-activating mutation	p53, thrombospondin 1, CD4^+^ cells	Genetically engineered mice	Kras2 activating mutation: mammary tumors	54–56, 59–61
						Disease regression factors: hematopoietic tumors, T cell acute lymphoblastic leukemia	
Wnt-1			Kras2-activating mutation		Genetically engineered mice	Mammary tumors	54
BCR-ABL				CD4^+^ cells	Genetically engineered mice	Pro-B cell leukemia	61
K-ras (G12D)	Increased Erk1/2, Akt1, Stat3/5, p38 phosphorylation				Genetically engineered mice	Lung and lymphatic tissue	56
ERα		Tamoxifen: increased Mucin4; increased MGMT; increased expression/ phosphorylation NF-κB pathway p50, RelB, and p65; decreased miR-375	Increased NF-κB pathway activity or decreased miR-375 following tamoxifen		Mucin4: MCF7/HER2–18 xenografts	Breast cancer	63–66, 76, 82
		Raloxifene: increased EGFR and Her2/Neu	Increased Her2/Neu following raloxifene		MGMT: breast cancer patients		
		Estrogen deprivation: up-regulated PDGF/Abl pathway			NF-κB pathway, miR-375, EGFR/HER2		
					PDGF/Abl:		
					MCF7 cell variants		

Development of such strategies might advance breast cancer prevention approaches. For example, tamoxifen is the only FDA-approved drug for chemoprevention of invasive breast cancer in premenopausal high-risk women; however, for every ten women who take tamoxifen, one to two will still develop invasive breast cancer. Both ERα^+^ and ERα^−^ disease is found in these cases.^62^ Similarly, while tamoxifen is reported to reduce development of noninvasive breast cancer (ductal carcinoma *in situ* (DCIS)), it does not absolutely prevent it.^74^ In fact, some estimate as many as 40% of tamoxifen-treated breast cancer patients relapse.^75^ These observations are not inconsistent with the prevalence of preexisting and acquired tamoxifen resistance in breast cancers. Because tamoxifen is already approved, it is logical to look for approaches that might improve tamoxifen efficacy, as any clinical trial would need evaluation against tamoxifen as the standard for premenopausal women. Postmenopausal women have the option of selecting raloxifene but, like tamoxifen, resistance to raloxifene also has been reported in experimental models.^76^ Raloxifene-resistant tumor cells demonstrated increased expression of both epidermal growth factor receptor (Egrf) (ERBB) and v-erb-b2 erythroblastic leukemia viral oncogene homolog 2, neuro/glioblastoma derived oncogene homolog (avian) (ERBB2). Experiments using pharmaceuticals targeted to the proteins encoded by these two RNAs, gefitinib for ERBB (epidermal growth factor receptor) and trastuzumab for ERBB2 (HER2/Neu) revealed that the upregulated ERBB2 was a critical secondary adaptation driving tumor growth but not ERBB. More recently, aromatase inhibitors (AIs) have been shown to have a role in breast cancer prevention for postmenopausal women.^77^–^81^ Here, experimental models have shown that the platelet-derived growth factor (PDGF)/Abl pathway is upregulated in response to estrogen deprivation.^82^ Women with AI-resistant breast cancer have been shown to respond better to the combination of tamoxifen with everolimus, an oral inhibitor of the mammalian target of rapamycin, compared with tamoxifen alone,^83^ illustrating the theme that preemptively targeting both primary disease-initiating stimuli as well as predicted secondary adaptations can improve therapeutic outcome.

Thinking of cancer prevention as biomodulation with attention not only to the initiating stimulus but also encompassing consequent alterations in cellular and biochemical pathways may provide a more robust approach to developing effective chemoprevention. State-of-the-art approaches to accomplish this may include the application of RNAseq, proteomics, and metabolomics to identify the multiplexed changes that occur as part of the process of biomodulation in epithelial tissue that has become preneoplastic. Validation of specific targets within what may be a multitude of changes will be challenging and difficult to accomplish utilizing only previous clinical trial designs. More personalized and intensive comprehensive studies that incorporate serial testing of the response to candidate agents and include the use of novel approaches for primary cell culture, both epithelial and stromal, as well as evaluation of systemic factors such as immune response, may be required. The use of genetically engineered mouse models are likely to facilitate development of this comprehensive approach as they provide a renewable source of material for testing the positive and negative predictive value and sensitivity of specific algorithms used for the development of new chemopreventive approaches targeting both the original lesion and the secondary changes that occur in the course of cancer development.
